# Exome sequencing revealed a novel homozygous variant in *TRMT61 A* in a multiplex family with atypical Cornelia de Lange Syndrome from Rwanda

**DOI:** 10.1186/s12920-025-02153-0

**Published:** 2025-05-13

**Authors:** Esther Uwibambe, Abdoulaye Yalcouyé, Elvis Twumasi Aboagye, Lettilia Xhakaza, Kalinka Popel, Norbert Dukuze, Thashi Bharadwaj, Carmen de Kock, Isabelle Schrauwen, Suzanne M. Leal, Leon Mutesa, Ambroise Wonkam

**Affiliations:** 1 Center for Human Genetics, College of Medicine and Health Sciences, University of Rwanda, Kigali, Rwanda; 2 Division of Human Genetics, Department of Medicine, Faculty of Health Sciences, University of Cape Town, Cape Town, South Africa; 3 Mckusick Nathans Institute and Department of Genetic Medicine, Johns Hopkins University, School of Medicine, Baltimore, MD 21205 USA; 4 Center for Statistical Genetics, Gertrude H. Sergievsky Center, and Department of Neurology, Columbia University Medical Center, New York, NY USA; 5 Department of Translational Neurosciences, University of Arizona College of Medicine –Phoenix, Phoenix, AZ 85004 USA; 6 Taub Institute for Alzheimer’s Disease and the Aging Brain, Columbia University Medical Center, New York, NY USA

**Keywords:** Cornelia de Lange Syndrome, Whole exome sequencing, *TRMT61 A*

## Abstract

**Background:**

In 30% of patients who exhibit the clinical profile of Cornelia de Lange Syndrome (CdLS), the genetic cause remains undetermined. This proportion tends to be higher in low-resource settings including Africa. We performed a molecular characterization of CdLS in a multiplex Rwandan family*.*

**Methods:**

After a clinical evaluation of two affected siblings, DNA isolated from peripheral whole blood of the affected patients and their parents underwent Exome Sequencing (ES). Sanger sequencing validated the variant segregating with CdLS. In silico predictive tools, protein modelling, and cell-based experiments using HEK293T cells were used to investigate the pathogenicity of the variant found.

**Results:**

We identified a family with two parents and their two offspring (male and female), who were referred for hearing impairment. The 17-year-old female presented bilateral profound hearing impairment with moderate hypertelorism, progressive visual impairment, and secondary amenorrhea. The 14-year-old male displayed intellectual disability and a bilateral profound hearing impairment with no noticeable facial dysmorphism. Following exome sequencing (ES) of DNA samples obtained from the four family members, we found that the siblings harbored a novel likely pathogenic homozygous missense variant in the *TRMT61 A* gene [NM_152307.3:c.665C > T p.(Ala222Val)] inherited from both heterozygous parents. I*n silico* analysis suggested that the variant substitutes a highly conserved amino acid, and 2-D structure modelling revealed a significant decrease in the stability of the protein. Cell-based experiment in HEK293T showed that the variant significantly affected the *TRMT61 A* protein localization which is thought to impact the mitochondrial and cytosolic functions.

**Conclusion:**

We reported a novel biallelic variant in *TRMT61 A,* [NM_152307.3:c.665C > T p.(Ala222Val)], which is associated with autosomal recessive atypical CdLS in a multiplex Rwandan family, the first report from Africa, and the second globally. The study emphasizes the need to expand the availability of ES for molecular characterization of rare diseases for the understudied genetically diverse population of Africa.

**Supplementary Information:**

The online version contains supplementary material available at 10.1186/s12920-025-02153-0.

## Introduction

Cornelia de Lange Syndrome (CdLS) is a rare genetic disease affecting < 1:2,000 and < 1:200,000 in the European Union and the US respectively [8]. Typical clinical signs include developmental and cognitive delay, growth restrictions, typical craniofacial dysmorphism including hirsutism [6]. Other features include limb and cardiac defects, hearing and vision impairment, and gastrointestinal and genitourinary disorders [6, 34]. Due to the extreme heterogeneity and phenotype spectrum, from mild to severe, the condition is classified as classic or non-classic, depending on the underlying genetic cause and the molecular algorithm used in the diagnosis [12].

The CdLS spectrum includes patients who exhibit the classic CdLS phenotype, in which the involved pathogenic gene variant plays a role in the cohesin functioning is either known or unknown (in which case the diagnosis is made based on clinical presentation if the implicated variant is unknown), as well as patients with a non-classic CdLS phenotype caused by a variant in a gene involved in cohesin functioning. Of note, both mildly and severely affected patients may exhibit classic and non-classic phenotypes of CdLS [12]. Therefore, CdLS is also genetically heterogeneous with up to seven associated genes in 70% of cases [12], namely, *NIPBL, SMC1 A, SMC3, RAD21, BRD4, HDAC8*, and *ANKRD11* [3, 6, 27, 11, 34]. In addition, the *TRMT61 A* gene has been reported to cause recessive CdLS in two siblings, but this association has not yet been well established by reports from and non-related additional affected families, or by functional investigations. However, CdLS is understudied on the African continent, with cases only reported from South Africa [33].

In this study, we performed a molecular characterization of an atypical CdLS in a multiplex Rwandan family using Exome Sequencing (ES), in silico prediction, protein modelling, and cell-based experiments HEK293 T. We described a novel homozygous likely pathogenic variant in *TRMT61 A,* [NM_152307.3:c.665 C > T p.(Ala222 Val)], in two affected siblings, the first report from Africa, and the second globally.

### Subjects, material, and methods

#### Participants and clinical assessments

Participants were enrolled at schools for the Deaf during community engagement activities across different regions of Rwanda, following a previously reported procedure [39]. A general practitioner, a medical geneticist, and an ear, nose, and throat (ENT) specialist reviewed the detailed personal history and medical records of affected individuals. Clinical examinations were performed, and pure tone audiometry (PTA) was conducted. The degree of HI was classified according to the American Speech-Language-Hearing Association (ASHA) [2].

### Molecular investigations

#### Exome sequencing

**Fig. 1:**
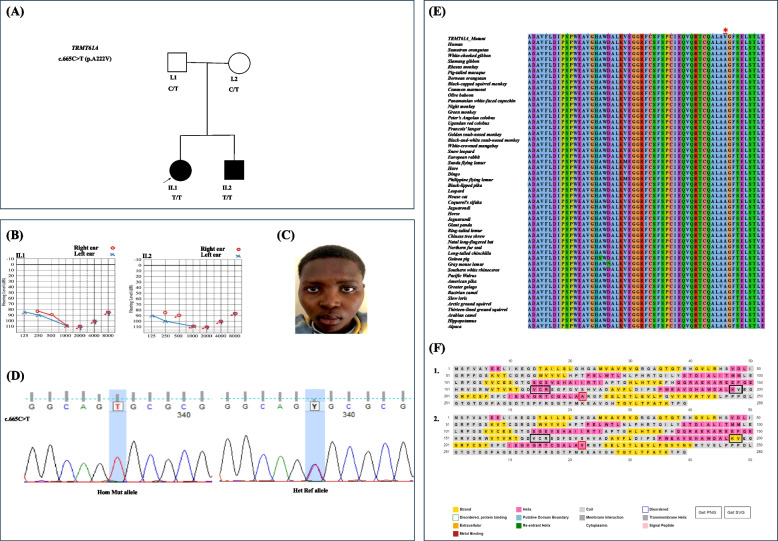
Pedigree, audiogram, chromatogram, amino acid conservation and protein modeling data of multiplex family RW.KG.SFS_.09. **A** Two-generation pedigree suggestive of an incomplete penetrance autosomal dominant mode of inheritance of the Cornelia de Lange syndrome. The black arrow indicates the proband. **B** Air conduction of pure tone audiometry of the affected brother and sister showing bilateral profound HI. **C** Photo of the affected female showing mild hypertelorism. **D** Chromatograms of Sanger sequencing of the missense *TRMT61 A* variant in the TRMT61 A gene *[NM_152307.3:c.665 C* > *T p.(Ala222 Val)]* and the reference allele (indicate the segregation on the pedigree). The position of the nucleotide change is highlighted in blue. **E** Evolutionary conservation of the *TRMT61 A*:* p.(Ala222 Val)* variant position (indicated by the red asterisk). **F** (1) Wild type, (2) Mutant, showing major structural changes (Black boxes). C/T, heterozygous mutant allele; T/T, homozygous mutant allele; Hom Mut, homozygous mutant for the variant allele; Het Ref, heterozygous reference allele

Following the exclusion of *GJB2* and *GJB6*, DNA from both sibling and their parent i.e., individuals I.1, I.2, II.1, and II.2 (Fig. 1A) underwent ES. Exomic libraries were prepared using the SureSelect Human All Exon V6 kit (Agilent Technologies, Santa Clara, CA, USA). Paired-end sequencing with 150 bp pair-end reads was conducted on a NovaSeq 6000 system (Illumina Inc., San Diego, CA, USA). Reads were aligned to the human reference genome hg38 using Burrows–Wheeler Aligner-MEM [14]. We performed joint variant calling via the Genome Analysis Toolkit v3 (GATK v3) [19]. Plink v1.9 was used to confirm the sex of each participant while familial relationships of all members were verified using Identity-by-Descent sharing (plinkv1.9) and the Kinship-based INference for Gwas (KING) algorithm [7, 17].

#### Annotation and filtering strategy

We performed variant filtering and annotation via ANNOVAR as previously described (29, 38). Variants were prioritized according to the assumed inheritance pattern (autosomal recessive or dominant), and variants with minor allele frequency (MAF) in all populations of the genome aggregation database (gnomAD, version v4.1.0). Only rare variants with a MAF below 0.005 for recessive and below 0.0005 for dominant inheritance, were retained. Additionally, known pathogenic or likely pathogenic HI variants listed in ClinVar were also investigated, regardless of their frequencies. After annotation, we employed in silico bioinformatics prediction scores from dbNSFP to assess the damaging effects of missense variants. In addition, we performed in silico predictions of synonymous and splicing variants using spliceAI and Human Splice Finder (HSF) tools to investigate the functional impact of potential intronic and splice variants.

Additional tools utilized to assess the pathogenicity of the variant included PolyPhen-2, SIFT, MutationTaster, DANN, CADD, TOPMed Freeze 9b, all of us and GERP +  + scores [1, 9, 21, 25, 26, 31]. Furthermore, variants were classified according to the American College of Medical Genetics (ACMG) [18].

#### Sanger sequencing

The variants identified underwent validation via direct sequencing and their segregation amongst available family members (Fig. 1). Furthermore, the candidate variant was screened in 100 healthy control individuals from Rwanda. Sequence-specific primer pairs targeting the variant region in exon 4 of the *TRMT61 A* gene were designed and evaluated using NCBI Primer BLAST. Selected primers were evaluated with IDT online primer optimization software. PCR followed by bi-directional Sanger sequencing were conducted using primer pair (Table S2) at the Division of Human Genetics, University of Cape Town, South Africa. FinchTV v1.4.0 [37] software was employed for the manual inspection of ABI file chromatograms, which were then aligned to the *TRMT61 A* reference sequence.

#### Evolutionary conservation

We aligned the sequence spanning the candidate variant found here with non-human species similar proteins to evaluate the conservation of the respective amino acid (aa). NCBI BLAST search against the non-redundant protein database was performed and the first 50 hits of different species were manually retrieved as FASTA files. Multiple sequence alignment (MSA) was performed using Clustal Omega [16] and the resulted alignments were viewed using Jalview version 2.11.4.1.

#### Secondary (2D) and three-dimensional (3D) protein modeling

The protein sequence (NP_689520.2) of TRMT61 A was retrieved from NCBI. The secondary structure was predicted on PSIPRED [4], and the 3D structure was modeled on SWISS-MODEL using AlphaFold structure (Q96 FX7) as a template [32]. The newly predicted structure was refined on the GalaxyWeb server [13]. Structure visualization and analysis were performed with Pymol software [30]. In addition, the I-mutant2.0 platform was used to investigate the stability of the mutant protein [5].

#### Expression pattern in human inner ear organoids and mouse

To investigate the expression pattern of *TRMT61 A *in vitro, we accessed the Human Inner Ear Organoids (scRNAseq and snRNAseq) and P1 mouse (scRNAseq) data from gene expression analysis resources (gEAR) [22]. The expression pattern in specific cells was analyzed.

### In vitro functional HEK293 T assay of the candidate *TRMT61 A* variant

#### Site-directed mutagenesis

Mammalian expression plasmids expressing open read frame of the human *TRMT61 A* with C-terminal tGFP-tag (#RG204071) was purchased from Origene and used as template for site directed mutagenesis (SDM) using our primers (Forward: CTGGCAGTGCGCGGCTTCTC and Reverse: CGCCTGGCATGTGCGTTGC) acquired from Integrated DNA Technologies (IDT, Coralville, IA, USA). SDM was performed in laboratory at the Department of Genetic Medicine, Johns Hopkins University, Baltimore, USA, using a protocol available upon request. Long read sequencing (LRS) of the plasmids was performed at Plasmidsaurus (Eugene, OR, USA) to confirm the variant of interest and assess any unwanted changes.

#### Cell culture, transfections, and visualization using confocal microscopy

Human embryonic kidney (HEK293 T) cells were cultured in complete Dulbecco’s Modified Eagle Medium (DMEM) (Thermo Fisher Scientific, Waltham, MA, USA) supplemented with 10% fetal bovine serum (Thermo Fisher Scientific, Waltham, MA, USA) and 1% Glutamine. Cells were cultured in a humidified incubator at 37 °C with 5% CO2. The HEK293 T cells were plated in 4-chamber dishes (density 5 × 10^4^ cells per mL), 18 h before transfection. Cells were transiently transfected using TurboFect transfection reagent according to the manufacturer’s instructions (Waltham, MA, USA), with 250 ng of plasmid (Empty, tGFP-only, tGFP-tagged wildtype and mutant of TRMT61 A). Cells were stained with Hoechst 33,342 (1:1000 dilution) dye for co-visualization of nuclear material. Live viewing (10 X and 40X) was performed at 48 h after transfection using Olympus FV3000RS Confocal Microscope. Images were visualized and processed using the FV31S-W viewer software.

#### Western blots

HEK293 T cells were incubated (density 6 × 10^5^ cells per mL) in 6-well plates, 18 h before transfection. Cells were transfected using TurboFect transfection reagent (Waltham, MA, USA) according to the manufacturer’s instructions, with 2000 ng of the WT or MT-*TRMT61 A* plasmid fused with the tGFP tag and non-transfected cells with no plasmid DNA. Total protein lysates were collected at 48 h for both genes using radioimmunoprecipitation assay (RIPA) buffer, with protease inhibitor cocktail (#P8340). Proteins were briefly sonicated and concentrations were checked using Qubit5 fluorometer. Then, 30ug total protein was denatured (85ºC, over 2 min) and loaded onto NovexTM WedgeWellTM 4–20% Tris–Glycine Gel. The gel electrophoresis was run at 100 V over 90 min. Proteins were transferred onto iBlot® 2 regular Stacks, nitrocellulose (NC), regular size (Invitrogen, Thermo Fischer, Scientific, Waltham, Massachusetts, USA). Membrane was stained with Revert TM 700 Total Protein Stain (LI-COR Biosciences, Lincoln, Nebraska). Then, the membrane was incubated with primary Mouse monoclonal turboGFP antibody (#TA150041; dilution 1:1000) and secondary antibody anti-Mouse (product #A11371; dilution 1:10,000; Thermo Fisher). Target signals were normalized to total protein signals to allow for a relative comparison of the wild type and mutant.

## Results

### Patients’ demographics and phenotypic descriptions

We identified a family of four (Fig. 1A), with two siblings referred for evaluation, as part of a study on HI in Rwanda. Upon clinical examination, the 17-year-old female presented bilateral profound HI (Fig. 1B), moderate hypertelorism, arched and thick eyebrows (Fig. 1C), progressive visual impairment, and secondary amenorrhea. Conversely, the 14-year-old affected male had intellectual disabilities, bilateral profound HI clinically confirmed by an audiogram, and low-set ears. There were no reported perinatal hazardous environmental exposures that could have contributed to their HI and other phenotypes. The phenotypic description that does not feature the typical facial phenotypes and the molecular analysis suggest a non-classical familial form of CdLS.

### Exome sequencing analysis

The average target region coverage was about 100X, with 96.30% of targeted regions covered at 10X or more. After applying various filtering criteria (described in the method section), the following variants were considered;

There was no variant detected in *GJB2* and *GJB6* genes, however, a homozygous missense variant, NM_152307.3:c.665 C > T p.(Ala222 Val), was called in the *TRMT61 A* gene by ES. The variant was validated by Sanger sequencing, where both parents were confirmed to be carriers of the variant (Fig. 1A and D). At the same time, the affected male and female harbored a homozygous form of the variant. The variant was not detected in any of the healthy controls (*n* = 100). The *in-silico* tools predicted the variant to be damaging (Supplementary Table 4). The variant was classified as likely pathogenic following the ACMG guidelines (PP1, PP3, PS3, PM2).

### Evolutionary conservation of amino acid

The position of the variant p.(Ala222 Val) was found to be 100% conserved across the top 50 hits resulted from the NCBI BLAST search (Fig. 1E). The region across which the amino acid is located is remarkably conserved and the variant could have led to the change in the structure and function of the protein.

### Secondary and three-dimensional (3D) protein modeling

Secondary structure analysis revealed that the mutant protein loses a helical structure located at ^164^VCR^166^ and gains a new beta-strand structure at ^197^VR^198^ (Fig. 1F). In addition, I-mutant 2 prediction showed that the variant decreases the stability of the protein compared to the wild type. However, the 3D structure did not reveal significant changes in the mutant compared to the wild type (Supplementary Fig. 5).

### In vitro functional HEK293 T assay

**Fig. 2:**
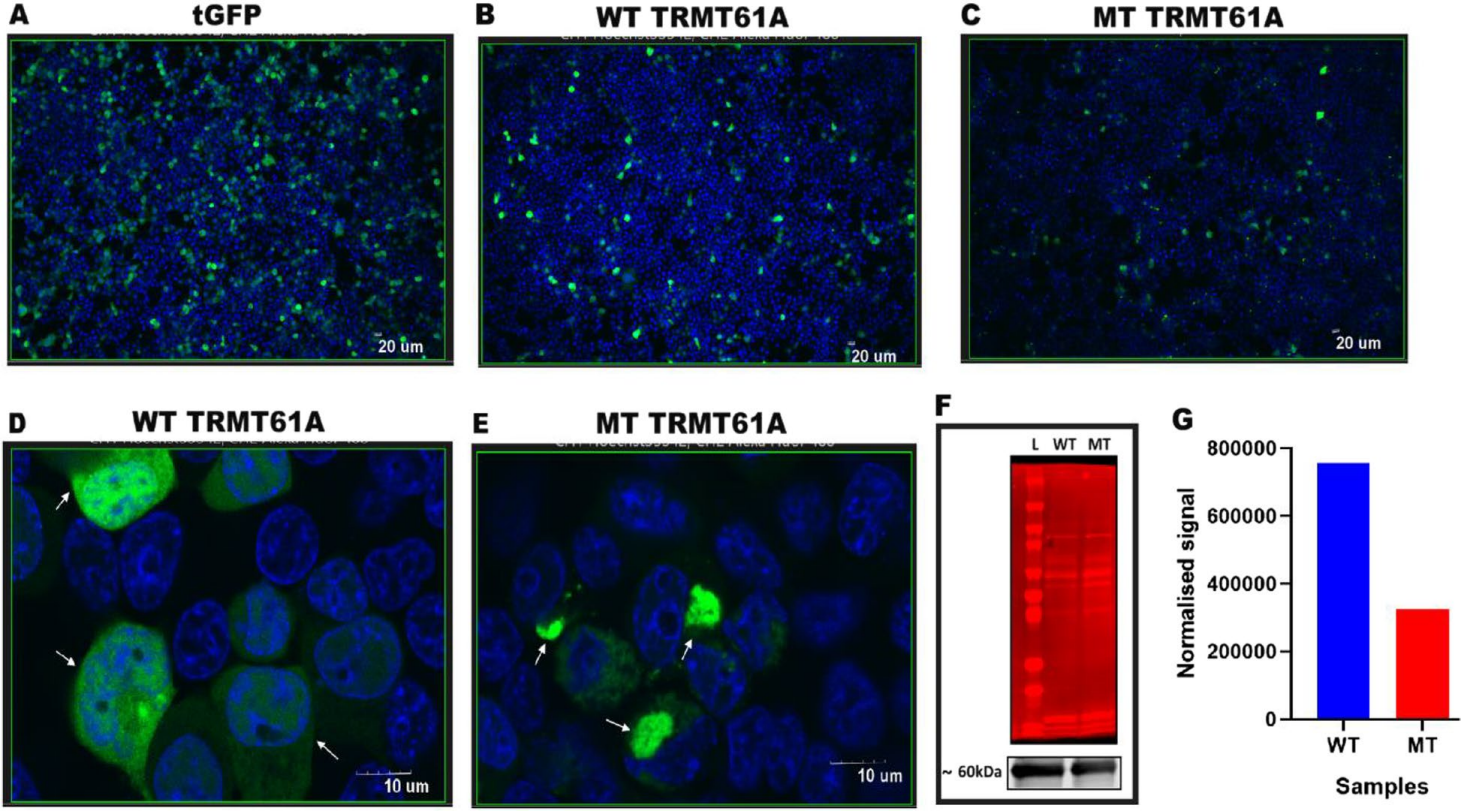
Functional analysis using HEK293 T cells. **A**-**C** Confocal microscopy images at 10X showing the co-localization of the nuclear material (blue) and tGFP fused TRMT61 A protein (green) of tGFP, wild type and mutant, respectively. **D** Wild type TRMT61 A *at* 40X showing a nuclear and cytoplasmic localisation (white arrows), **E** Mutant TRMT61 A at 40X showing a predominant nuclear accumulation (white arrows), **F**-**G** Western blot analysis showing the decreased level of the normalised target signal in the mutant protein compared to the wild type. L: stands for the ladder, WT: wild type and MT: mutant

Live viewing confirmed the successful transfection and expression of tGFP, WT and mutant (Fig. 2A-C). However, the fluorescence signal was lower in mutant protein compared to WT. In addition, the WT tGFP fused protein seems to localise in the nucleus and cytoplasm as expected (Fig. 2D). On the other hand, the mutant protein appears to accumulate and localise mainly in the nucleus (Fig. 2E). Furthermore, western blot analysis showed that the specific bands were at ~ 60 kDa for WT and mutant, however, the normalized target signal was lower in mutant protein (Fig. 2F, G, Supplementary Figure S2 and Supplementary Table 3), consistent with the finding of the confocal microscopy analysis.

### Expression pattern in Human inner ear and mouse

**Fig. 3:**
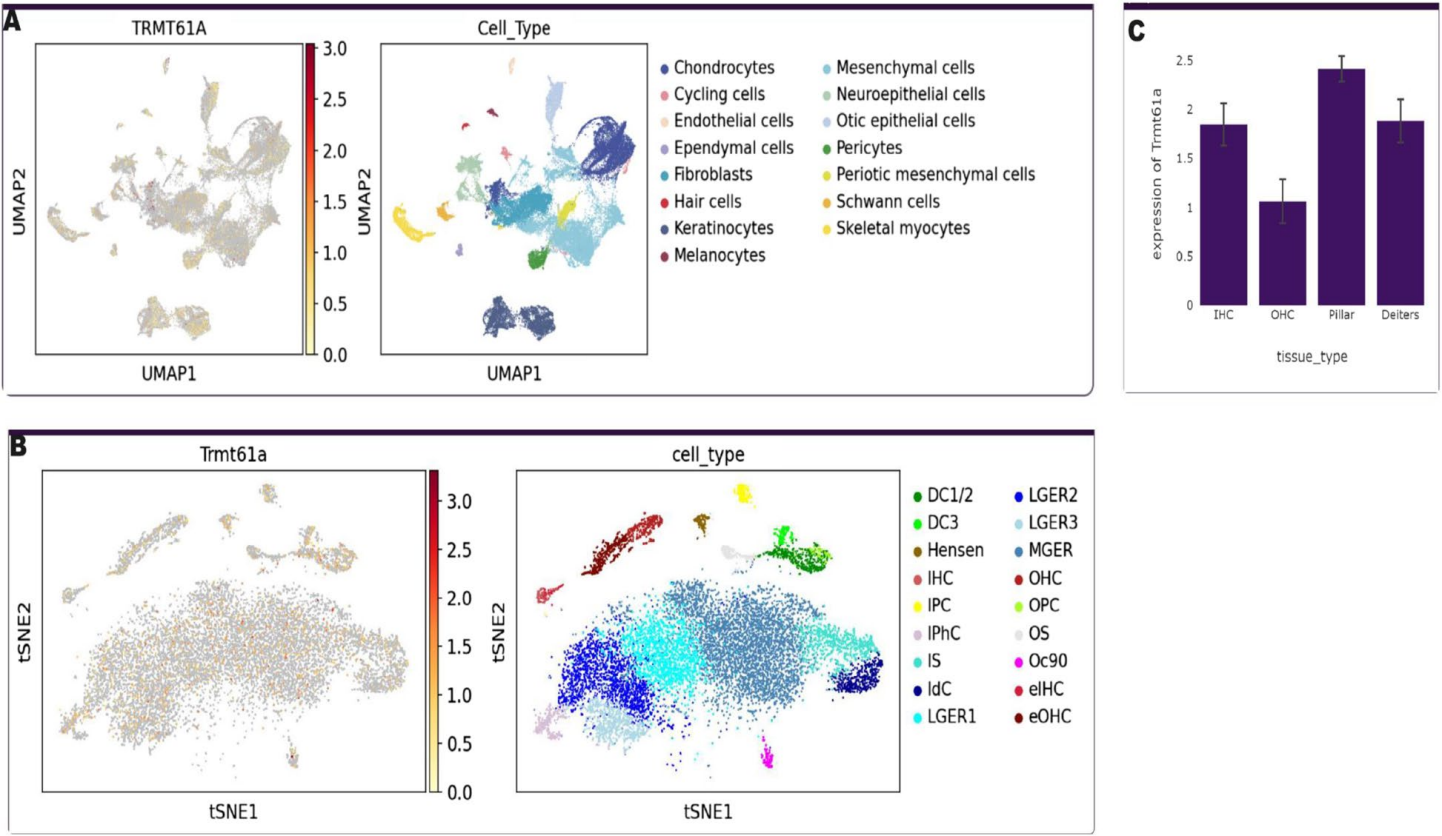
Expression profile of TRMT61 A. **A** Human inner ear organoids. **B**-**C** P1 mouse

Expression analysis showed that *TRMT61 A* is expressed in both human inner ear organoids and mouse RNAseq data including the hair and supporting cells (Fig. 3A-C).

## Discussion

Molecular characterization of rare diseases such as CdLS have been rarely reported in Sub-Saharan Africa. Using ES in a multiplex family with two cases, we describe an autosomal recessive case of atypical form of CdLS due to a novel homozygous missense variant, c.665 C > T p.(Ala222 Val), in the *TRMT61 A* gene. We reported clinical evidence of two affected sibling, in silico pathogenic scores using PolyPhen-2 and Mutation Taster, and functional assays in HEK293 T cells which found that the NM_152307.3:c.665 C > T p.(Ala222 Val) variant significantly alter TRMT61 A protein localization, which is thought to impair both mitochondrial and cytosolic functions. Taken together, these findings suggest that the variant is likely pathogenic and may have a causal relationship with the clinical phenotypes observed in the two siblings.

The affected Rwandan brother and sister can still be classified as atypical cases of CdLS, although they lack many of the classical characteristics typically associated with the condition. Specifically, they do not exhibit the hallmark features such as significant growth and cognitive delays, hirsutism, distinct facial dysmorphism, or limb malformations—particularly of the upper limbs. However, both siblings presented with speech impairment and bilateral profound HI, symptoms commonly observed in CdLS. Additionally, the affected brother presented with intellectual disability and low-set ears while the affected sister displayed arched and thick eyebrows, hypertelorism, progressive vision loss, and secondary amenorrhea—features that have also been documented in individuals with CdLS. The carrier parents were seemingly healthy and did not show any phenotypes.

In contrast, two Palestinian sisters described by Sharif in 2020 exhibited a more typical CdLS phenotype. Their clinical presentation included craniofacial abnormalities such as microcephaly, synophrys, anteverted nares, and micrognathia, as well as pronounced speech and hearing impairments. Furthermore, they experienced growth and cognitive retardation, hirsutism, upper limb anomalies, and gastrointestinal complications. These sisters were found to carry compound heterozygous variants—an in-frame deletion and a missense mutation—in the *TRMT61 A* gene, consistent with an autosomal recessive inheritance pattern. Their parents were each heterozygous carriers of the respective variants: [c.478_483 delCGCACC:p.(R160_T161 del)] and [c.323G > T:p.(C108 F)]. Therefore, the Rwandan siblings represent only the second reported instance worldwide of CdLS associated with likely pathogenic variants in *TRMT61 A,* broadening the phenotypic spectrum of *TRMT61 A*-related CdLS.

*PolyPhen-2* and *mutation taster* computational predictive tools predicted that the variant, c.665 C > T p.(Ala222 Val), is damaging (Supplementary Table S4). The decreased expression and mis-localization of mutant TRMT61 A in cell-based experiments suggest that the predominantly nuclear localisation caused by the variant could impact the mitochondrial and cytosolic functions of TRMT61 A [15, 28]. The high conservation of the amino acid across several species (Fig. 1E) could explain the fault in the protein’s structure and function when mutated, hence the CdLS phenotype.

TRMT61 A gene exists in a heterotetramer complex of 2 TRMT61 A subunits and 2 TRMT6 subunits. The two genes work together to regulate gene expression by ensuring the necessary post-transcription modification of tRNA and mRNA [10, 35]. *TRMT61 A* encodes for the tRNA-methyltransferase enzyme, which converts N1 adenine at position 58 into N_1_-methyladenine 58 (m1 A58) by catalyzing the methylation reaction that transfers a methyl group from the methyl donor (S-adenosyl-methionine) to N1 of adenine 58 in the initiator methionyl-tRNA [23]. Studies have linked defects in tRNA modification processes to various human diseases such as cancer, type 2 diabetes, neurological disorders, and mitochondrial-linked disorders, underscoring the importance of genes like *TRMT61 A* in cellular physiology [36]. Studies are yet to clarify the specific relationship between *TRMT61 A* and the other genes known to be the main contributors to CdLS*,* such as *NIPBL* and *SMC1 A*; however, *TRMT61 A* is one of the accessory genes misregulated when these genes are mutated in CdLS patients [11]*.* Coupled with this, *TRMT61 A* and *NIPBL* genes both play a role in gene expression and genome stability, which plausibly could partly explain its association with CdLS [20, 24].

## Conclusion

We reported a novel biallelic variant in the *TRMT61 A* gene [NM_152307.3:c.665 C > T p.(Ala222 Val)] which is associated with atypical CdLS in two sibling from a multiplex Rwandan family, the first report from Africa, and the second globally. The study emphasized the need to expand availability of whole exome sequencing for molecular characterization of rare diseases for understudied genetically diverse populations of Africa.

## Supplementary Information

Supplementary Material 1: Supplementary Figure 1: Superimposed three-dimensionalstructures of TRMT61 A.Greenand light blueshowing no significant structural changes. Supplementary Figure S2: TRMT61 A_orignal Western blots

## Data Availability

No datasets were generated or analysed during the current study.

## Abbreviations

TRMT61 A: TRNA methyl transferase 61A
TRMT6: TRNA methyl transferase 6
NIPBL: Nippled-B-Like
SMC1 A: Structure maintenance of chromosome 1A
SMC3: Structure maintenance of chromosome 3
RAD21: RAD21 cohesin complex component
BRD4: Bromodomain-conaining protein 4
HDAC8: Histone deacetylase 8
ANKRD11: Ankyrin repeat domain-conaining protein 11
DNA: Deoxyribonucleic acid
HEK293 T: Human embryonic kidney 293
ES: Exome sequencing
HI: Hearing impairment
GJB2: Gap junction beta-2
GJB6: Gap junction beta-6
GWAS: Genome-wide association studies
ANNOVAR: ANNOtate VARiation
CADD: Combined Annotation Dependent Depletion
SIFT: Sorting Intolerant From Tolerant
DANN: Deleterious Annotation of Genetic Variants using Neural Networks
GERP: Genomic Evolutionary Rate Profiling
TOPMed: Trans-Omics for Precision Medicine
NCBI: National Center for Biotechnology Information
BLAST: Basic Local Alignment Search Tool
IDT: Integrated DNA Technologies
PCR: Polymerase chain reaction
ABI: Applied Biosystems genetic analyzer
FASTA: Fast-All
PSIPRED: PSI-blast based secondary structure PREDiction
SWISS: Swiss-Prot
SDM: Site-Directed Mutagenesis
WT: Wild type
MT: Mitochondria
NM: Reference sequence nucleotide
ACMG: American College of Medical Genetics and Genomics
PP: Pathogenic Supporting
PS: Pathogenic Strong
PM: Pathogenic Moderate
RNA: Ribonucleic Acid
IRB: Institutional Review Board
US: United States of America

## References

[1] Adzhubei IA, Schmidt S, Peshkin L, Ramensky VE, Gerasimova A, Bork P, Kondrashov AS, Sunyaev SR. A method and server for predicting damaging missense mutations. Nat Methods. 2010;7(4):248–9. 10.1038/nmeth0410-248. 20354512 10.1038/nmeth0410-248 PMC2855889

[2] ASHA. Uses and abuses of hearing loss classification Degree of Hearing Loss. 1981. https://www.Researchgate.Net/Publication/16145943_Uses_and_abuses_of_hearing_loss_classification; American Speech-Language-Hearing Association.https://www.asha.org/public/hearing/degree-of-hearing-loss/. Accessed 11 Dec 2024

[3] Boyle M, i., Jespersgaard, C., Brøndum-Nielsen, K., Bisgaard, A.-M., & Tümer, Z. Cornelia de Lange syndrome. Clin Genet. 2015;88(1):1–12. 10.1111/cge.12499. 25209348 10.1111/cge.12499

[4] Buchan DWA, Jones DT. The PSIPRED Protein Analysis Workbench: 20 years on. Nucleic Acids Res. 2019;47(W1):W402–7. 10.1093/nar/gkz297. 31251384 10.1093/nar/gkz297 PMC6602445

[5] Capriotti E, Fariselli P, Casadio R. I-Mutant2.0: Predicting stability changes upon mutation from the protein sequence or structure. Nucleic Acids Res. 2005;33(Web Server issue):W306–10. 10.1093/nar/gki375. 15980478 10.1093/nar/gki375 PMC1160136

[6] Cascella M, Muzio MR. Cornelia de Lange Syndrome. In StatPearls. StatPearls Publishing. 2024. PMID: 32119471; Bookshelf ID: NBK554584.http://www.ncbi.nlm.nih.gov/books/NBK554584/. 32119471

[7] Chang CC, Chow CC, Tellier LC, Vattikuti S, Purcell SM, Lee JJ. Second-generation PLINK: Rising to the challenge of larger and richer datasets. GigaScience. 2015;4:7. 10.1186/s13742-015-0047-8. 25722852 10.1186/s13742-015-0047-8 PMC4342193

[8] Danese E, Lippi G. Rare diseases: The paradox of an emerging challenge. Ann Transl Med. 2018;6(17):329. 10.21037/atm.2018.09.04. 30306068 10.21037/atm.2018.09.04 PMC6174191

[9] Davydov EV, Goode DL, Sirota M, Cooper GM, Sidow A, Batzoglou S. Identifying a High Fraction of the Human Genome to be under Selective Constraint Using GERP++. PLoS Comput Biol. 2010;6(12):e1001025. 10.1371/journal.pcbi.1001025. 21152010 10.1371/journal.pcbi.1001025 PMC2996323

[10] Finer-Moore J, Czudnochowski N, O’Connell JD, Wang AL, Stroud RM. Crystal Structure of the Human tRNA m 1A58 Methyltransferase–tRNA3Lys Complex: Refolding of Substrate tRNA Allows Access to the Methylation Target. J Mol Biol. 2015;427(24):3862–76. 10.1016/j.jmb.2015.10.005. 26470919 10.1016/j.jmb.2015.10.005 PMC4663122

[11] Imène Boudaoud. NIPBL et le complexe cohésine lient l’organisation 3D des gènes à la régulation transcriptionnelle. 2018. https://corpus.ulaval.ca/bitstreams/01b6740a-6e5c-47ae-89d8-4cab9d7c94b9/download. Accessed 11 Dec 2024

[12] Kline AD, Moss JF, Selicorni A, Bisgaard A-M, Deardorff MA, Gillett PM, Ishman SL, Kerr LM, Levin AV, Mulder PA, Ramos FJ, Wierzba J, Ajmone PF, Axtell D, Blagowidow N, Cereda A, Costantino A, Cormier-Daire V, FitzPatrick D, Hennekam RC. Diagnosis and management of Cornelia de Lange syndrome: First international consensus statement. Nat Rev Genet. 2018;19(10):649–66. 10.1038/s41576-018-0031-0. 29995837 10.1038/s41576-018-0031-0 PMC7136165

[13] Ko J, Park H, Heo L, Seok C. GalaxyWEB server for protein structure prediction and refinement. Nucleic Acids Res. 2012;40(Web Server issue):W294–7. 10.1093/nar/gks493. 22649060 10.1093/nar/gks493 PMC3394311

[14] Li H, Durbin R. Fast and accurate long-read alignment with Burrows-Wheeler transform. Bioinformatics (Oxford, England). 2010;26(5):589–95. 10.1093/bioinformatics/btp698. 20080505 10.1093/bioinformatics/btp698 PMC2828108

[15] Li X, Xiong X, Zhang M, Wang K, Chen Y, Zhou J, Mao Y, Lv J, Yi D, Chen X-W, Wang C, Qian S-B, Yi C. Base-resolution mapping reveals distinct m1A methylome in nuclear- and mitochondrial-encoded transcripts. Mol Cell. 2017;68(5):993-1005.e9. 10.1016/j.molcel.2017.10.019. 29107537 10.1016/j.molcel.2017.10.019 PMC5722686

[16] Madeira F, Madhusoodanan N, Lee J, Eusebi A, Niewielska A, Tivey ARN, Lopez R, Butcher S. The EMBL-EBI Job Dispatcher sequence analysis tools framework in 2024. Nucleic Acids Res. 2024;52(W1):W521–5. 10.1093/nar/gkae241. 38597606 10.1093/nar/gkae241 PMC11223882

[17] Manichaikul A, Mychaleckyj JC, Rich SS, Daly K, Sale M, Chen W-M. Robust relationship inference in genome-wide association studies. Bioinformatics. 2010;26(22):2867–73. 10.1093/bioinformatics/btq559. 20926424 10.1093/bioinformatics/btq559 PMC3025716

[18] Masson E, Zou W-B, Génin E, Cooper DN, Le Gac G, Fichou Y, Pu N, Rebours V, Férec C, Liao Z, Chen J-M. Expanding ACMG variant classification guidelines into a general framework. Hum Genomics. 2022;16:31. 10.1186/s40246-022-00407-x. 35974416 10.1186/s40246-022-00407-x PMC9380380

[19] McKenna A, Hanna M, Banks E, Sivachenko A, Cibulskis K, Kernytsky A, Garimella K, Altshuler D, Gabriel S, Daly M, DePristo MA. The Genome Analysis Toolkit: A MapReduce framework for analyzing next-generation DNA sequencing data. Genome Res. 2010;20(9):1297–303. 10.1101/gr.107524.110. 20644199 10.1101/gr.107524.110 PMC2928508

[20] Motorin Y, Helm M. TRNA Stabilization by Modified Nucleotides. Biochemistry. 2010;49(24):4934–44. 10.1021/bi100408z. 20459084 10.1021/bi100408z

[21] Ng PC, Henikoff S. Predicting Deleterious Amino Acid Substitutions. Genome Res. 2001;11(5):863–74. 10.1101/gr.176601. 11337480 10.1101/gr.176601 PMC311071

[22] Orvis J, Gottfried B, Kancherla J, Adkins RS, Song Y, Dror AA, Olley D, Rose K, Chrysostomou E, Kelly MC, Milon B, Matern MS, Azaiez H, Herb B, Colantuoni C, Carter RL, Ament SA, Kelley MW, White O, Hertzano R. gEAR: Gene Expression Analysis Resource portal for community-driven, multi-omic data exploration. Nat Methods. 2021;18(8):843–4. 10.1038/s41592-021-01200-9. 34172972 10.1038/s41592-021-01200-9 PMC8996439

[23] Ozanick S, Krecic A, Andersland J, Anderson JT. The bipartite structure of the tRNA m1A58 methyltransferase from S. cerevisiae is conserved in humans. RNA. 2005;11(8):1281–90. 10.1261/rna.5040605. 16043508 10.1261/rna.5040605 PMC1370811

[24] Peters J-M, Tedeschi A, Schmitz J. The cohesin complex and its roles in chromosome biology. Genes Dev. 2008;22(22):3089–114. 10.1101/gad.1724308. 19056890 10.1101/gad.1724308

[25] Quang D, Chen Y, Xie X. DANN: A deep learning approach for annotating the pathogenicity of genetic variants. Bioinformatics. 2015;31(5):761–3. 10.1093/bioinformatics/btu703. 25338716 10.1093/bioinformatics/btu703 PMC4341060

[26] Rentzsch P, Witten D, Cooper GM, Shendure J, Kircher M. CADD: Predicting the deleteriousness of variants throughout the human genome. Nucleic Acids Res. 2019;47(Database issue):D886–94. 10.1093/nar/gky1016. 30371827 10.1093/nar/gky1016 PMC6323892

[27] Revenkova E, Focarelli ML, Susani L, Paulis M, Bassi MT, Mannini L, Frattini A, Delia D, Krantz I, Vezzoni P, Jessberger R, Musio A. Cornelia de Lange syndrome mutations in SMC1A or SMC3 affect binding to DNA. Hum Mol Genet. 2009;18(3):418–27. 10.1093/hmg/ddn369. 18996922 10.1093/hmg/ddn369 PMC2722190

[28] Safra M, Sas-Chen A, Nir R, Winkler R, Nachshon A, Bar-Yaacov D, Erlacher M, Rossmanith W, Stern-Ginossar N, Schwartz S. The m1A landscape on cytosolic and mitochondrial mRNA at single-base resolution. Nature. 2017;551(7679):251–5. 10.1038/nature24456. 29072297 10.1038/nature24456

[29] Schrauwen I, Liaqat K, Schatteman I, Bharadwaj T, Nasir A, Acharya A, Ahmad W, Van Camp G, Leal SM. Autosomal Dominantly Inherited GREB1L Variants in Individuals with Profound Sensorineural Hearing Impairment. Genes (Basel). 2020;23;11(6):687. 10.3390/genes11060687 PMC7349314 32585897

[30] Schrödinger. *PyMOL | pymol.org*. PyMOL by Schrödinger. https://pymol.org/. 2019. Accessed 20 Nov 2024

[31] Schwarz JM, Rödelsperger C, Schuelke M, Seelow D. MutationTaster evaluates disease-causing potential of sequence alterations. Nat Methods. 2010;7(8):575–6. 10.1038/nmeth0810-575. 20676075 10.1038/nmeth0810-575

[32] Schwede T, Kopp J, Guex N, Peitsch MC. SWISS-MODEL: An automated protein homology-modeling server. Nucleic Acids Res. 2003;31(13):3381–5 (https://www.ncbi.nlm.nih.gov/pmc/articles/PMC168927/). 12824332 10.1093/nar/gkg520 PMC168927

[33] Seymour H, Feben C, Nevondwe P, Kerr R, Spencer C, Mudau M, Honey E, Lombard Z, Krause A, Carstens N. Mutation profiling in South African patients with Cornelia de Lange syndrome phenotype. Mol Genet Genomic Med. 2024;12(1):e2342. 10.1002/mgg3.2342. 38284454 10.1002/mgg3.2342 PMC10785556

[34] Sharif FA. An Autosomal Recessive form of Cornelia de Lange Syndrome Due to Mutations in TRMT61A Gene: A Case Report. J Adv Med Medical Res. 2020;32(24):327–331. 10.9734/jammr/2020/v32i2430785.

[35] Su Z, Monshaugen I, Wilson B, Wang F, Klungland A, Ougland R, Dutta A. TRMT6/61A-dependent base methylation of tRNA-derived fragments regulates gene-silencing activity and the unfolded protein response in bladder cancer. Nat Commun. 2022;13(1):1. 10.1038/s41467-022-29790-8. 35444240 10.1038/s41467-022-29790-8 PMC9021294

[36] Torres AG, Batlle E, Ribas De Pouplana L. Role of tRNA modifications in human diseases. Trends Mol Med. 2014;20(6):306–14. 10.1016/j.molmed.2014.01.008. 24581449 10.1016/j.molmed.2014.01.008

[37] Treves DS. Review of Three DNA Analysis Applications for Use in the Microbiology or Genetics Classroom. J Microbiol Biol Educ. 2010;11(2):186–7. 10.1128/jmbe.v11i2.205. 23653728 10.1128/jmbe.v11i2.205 PMC3577175

[38] Wang K, Li M, Hakonarson H. ANNOVAR: functional annotation of genetic variants from high-throughput sequencing data. Nucleic Acids Res. 2010;38(16):e164. 10.1093/nar/gkq603 PMC2938201 20601685

[39] Wonkam A, Noubiap JJN, Djomou F, Fieggen K, Njock R, Toure GB. Aetiology of childhood hearing loss in Cameroon (sub-Saharan Africa). Eur J Med Genet. 2013;56(1):20–5. 10.1016/j.ejmg.2012.09.010. 23085303 10.1016/j.ejmg.2012.09.010

