# Supplementary material for: Exome sequencing revealed a novel homozygous variant in TRMT61 A in a multiplex family with atypical Cornelia de Lange Syndrome from Rwanda
Source: BMC Med Genomics. 2025 May 13;18:85. doi: 10.1186/s12920-025-02153-0 (PMC12070710; doi:10.1186/s12920-025-02153-0)
Supplement: Supplementary file 1 — Supplementary Material 1: Supplementary Figure 1: Superimposed three-dimensionalstructures of TRMT61 A.Greenand light blueshowing no significant structural changes. Supplementary Figure S2: TRMT61 A_orignal Western blots [file 12920_2025_2153_MOESM1_ESM.zip › Supplementary tables and figure_CdLS_Case report.docx]

**Supplementary materials**

**Supplementary Table 1: Primer sequences for GJB2 and GJB6 coding region amplification**

| **Gene** | **Primer** | **Primer sequence** | **Product size** |
| --- | --- | --- | --- |
| *GJB2* | F4 | 5’ -GCTTACCCAGACTCAGAGAAG-3’ | 900 |
|  | R1 | 5’-CTTAATCTAACAACTGGGCAATGC-3’ |  |
| *GJB6* | CDF | 5’-TTGGCTTCAGTATGTAATATCACC-3’ | 990 |
|  | CDR | 5’-TCATTTACAAACTCTTCAGGCTACAG-3’ |  |

**Supplementary Table 2: Allele-specific primers for the identified TRMT61A variant**

| **Primer name (Forward/Reverse)** | **Sequence (5'->3')** | **Nucleotides** | **Tm /˚C** | **Product size** | **PCR conditions** |
| --- | --- | --- | --- | --- | --- |
| *TRMT61A*_Ex_4_FF | TGGGCCAAGTGAGACAATGG | **20** | 60.25 | 774 | **Denaturation**   - 95°C, 3 min   **Annealation and extension (**35 cycles**):**   - 95°C, 30 sec - 60°C, 30 sec - 72°C, 1 min   **Final extension:**   - 72°C, 5 min   **Hold at 12°C** |
| *TRMT61A*_Ex_4_RR | CTGGACAGGAATGGCTGAGG | **20** | 60.11 |  |  |

**Supplementary Table 3: Western blot analysis data**

| Channel | Name | Total signal | Normalization factor |
| --- | --- | --- | --- |
| 700 | WT | 4320000 | 0.776978417 |
| 700 | MT | 5560000 | 1 |
|  |  |  |  |
| Channel | **Name** | **Target signal** | **Normalized signal** |
| 800 | WT | 588000 | 756778 |
| 800 | MT | 325000 | 325000 |

**Supplementary Table 4: In silico computational prediction and allele frequencies of the TRMT61A (NM_152307.3:c.665C>T) variant**

| **Gene** | **gnomAD** | **Bravo** | **CADD** | **All of Us** | **PolyPhen-2** | **SIFT** | **Mutation taster** | **dbSNP MAF** |
| --- | --- | --- | --- | --- | --- | --- | --- | --- |
| ***TRMT61A*** | 0.0009918 | 0.0010729 | 19.99 | 0.000913 | Possibly Damaging | Tolerated | Disease-causing | 0.00049 |


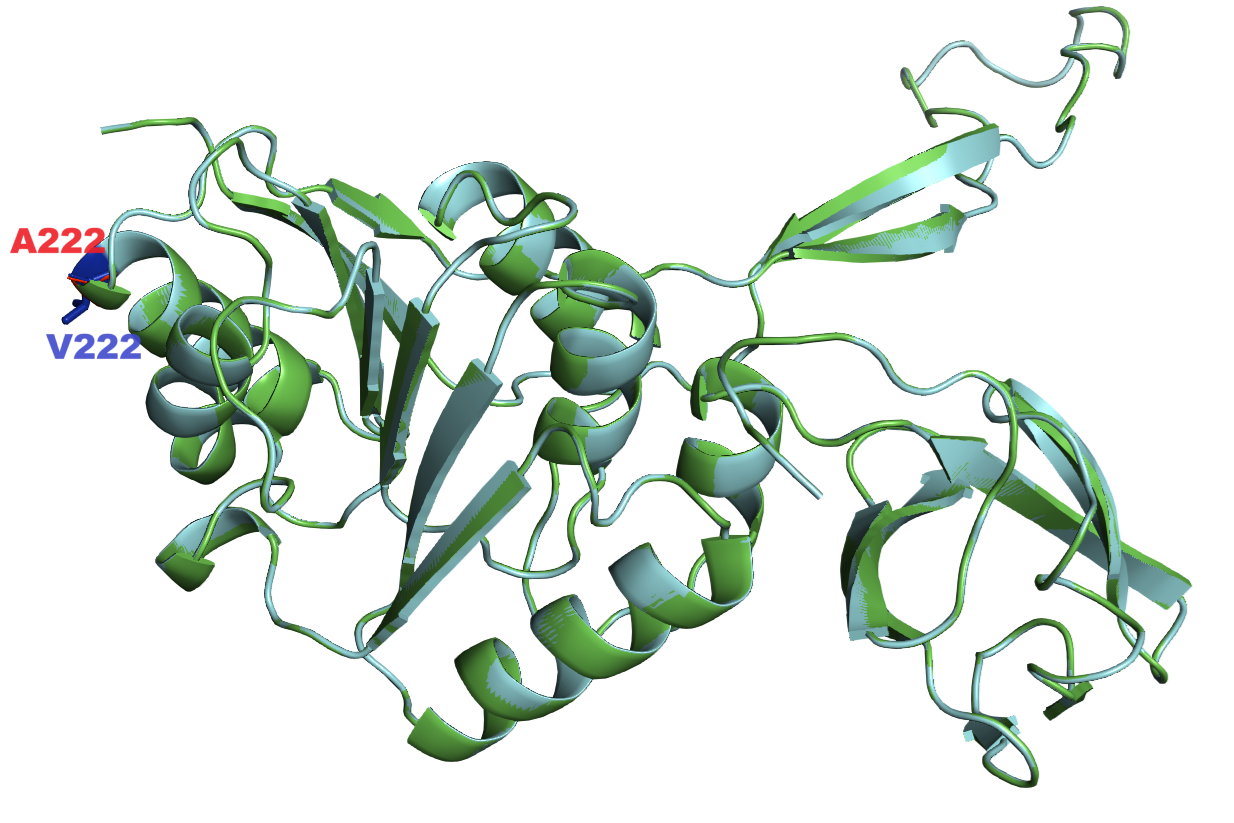


**Supplementary Figure 1: Superimposed three-dimensional (3D) structures of TRMT61A.** A) Green (wild type) and light blue (mutant) showing no significant structural changes.
