# Supplementary figures and images for: Exome sequencing revealed a novel homozygous variant in TRMT61 A in a multiplex family with atypical Cornelia de Lange Syndrome from Rwanda
Source: BMC Med Genomics. 2025 May 13;18:85. doi: 10.1186/s12920-025-02153-0 (PMC12070710; doi:10.1186/s12920-025-02153-0)

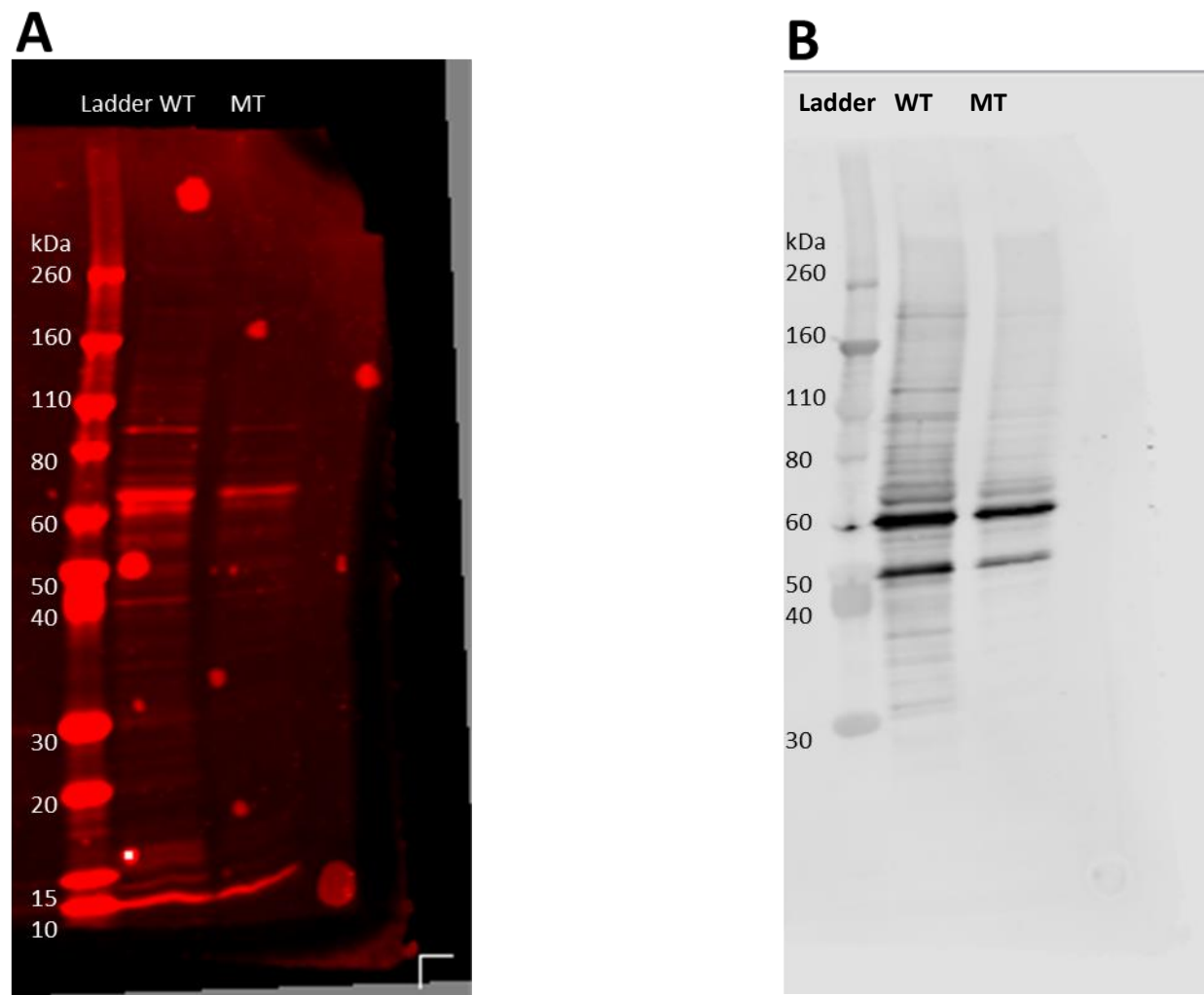

**Figure S2:** Unprocessed Western blot images: A) Total protein staining, B) Target protein

Supplement: Supplementary file 1 — Supplementary Material 1: Supplementary Figure 1: Superimposed three-dimensionalstructures of TRMT61 A.Greenand light blueshowing no significant structural changes. Supplementary Figure S2: TRMT61 A_orignal Western blots [file 12920_2025_2153_MOESM1_ESM.zip › FigS2. TRMT61A_original Western blots.pdf]
